# Self-Consistent Implementation of Kohn–Sham Adiabatic Connection Models with Improved Treatment of the Strong-Interaction Limit

**DOI:** 10.1021/acs.jctc.2c00352

**Published:** 2022-09-12

**Authors:** Szymon Śmiga, Fabio Della Sala, Paola Gori-Giorgi, Eduardo Fabiano

**Affiliations:** † Institute of Physics, Faculty of Physics, Astronomy and Informatics, Nicolaus Copernicus University in Toruń, ul. Grudziądzka 5, 87-100 Toruń, Poland; ‡ Institute for Microelectronics and Microsystems (CNR-IMM), Campus Unisalento, Lecce, Via Monteroni 73100, Italy; § Center for Biomolecular Nanotechnologies, Istituto Italiano di Tecnologia, Via Barsanti 14, Arnesano, Lecce 73010, Italy; ∥ Department of Chemistry & Pharmaceutical Sciences and Amsterdam Institute of Molecular and Life Sciences (AIMMS), Faculty of Science, Vrije Universiteit, De Boelelaan 1083, 1081HV Amsterdam, The Netherlands

## Abstract

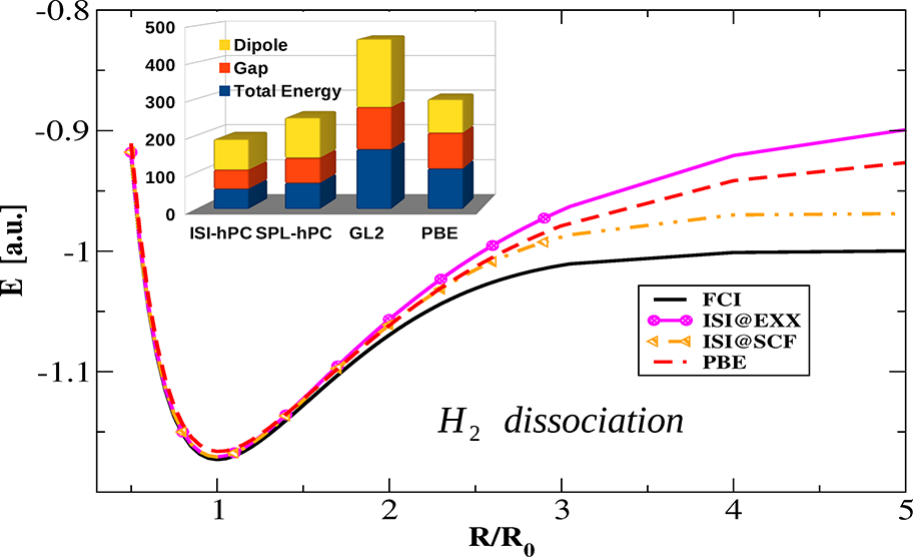

Adiabatic connection models (ACMs), which interpolate between the limits of weak and strong interaction, are powerful tools to build accurate exchange–correlation functionals. If the exact weak-interaction expansion from the second-order perturbation theory is included, a self-consistent implementation of these functionals is challenging and still absent in the literature. In this work, we fill this gap by presenting a fully self-consistent-field (SCF) implementation of some popular ACM functionals. While using second-order perturbation theory at weak interactions, we have also introduced new generalized gradient approximations (GGAs), beyond the usual point-charge-plus-continuum model, for the first two leading terms at strong interactions, which are crucial to ensure robustness and reliability. We then assess the SCF–ACM functionals for molecular systems and for prototypical strong-correlation problems. We find that they perform well for both the total energy and the electronic density and that the impact of SCF orbitals is directly connected to the accuracy of the ACM functional form. For the H_2_ dissociation, the SCF–ACM functionals yield significant improvements with respect to standard functionals also thanks to the use of the new GGAs for the strong-coupling functionals.

## Introduction

Kohn–Sham (KS)^1^ density functional theory (DFT) is the most used electronic structure computational approach for molecular and solid-state systems.^2−4^ Its accuracy depends on the choice of the approximation for the exchange–correlation (XC) functional^5−7^ which, at the highest-rung of the Jacob’s ladder,^8^ involves all the occupied and virtual KS orbitals as well as the eigenvalues. Then, the XC approximation is no more an explicit functional of the density and, to stay within the pure KS formalism, the optimized effective potential (OEP) method^9,10^ must be employed. Early OEP approaches included exact-exchange (EXX) and approximated the correlation using the second-order Görling–Levy perturbation theory (GL2).^11^ However, this led to a large overestimation of correlation effects and to convergence problems.^12−18^

Actually two different main approaches have been explored to solve this issue: going beyond the second-order approximation^19−26^ or using a semicanonical transformation.^12,13,18^ Another possible path is the adiabatic connection (AC) formalism^27−29^ which is a general, powerful tool for the development of XC functionals. For several decades, it has been used to justify the introduction of hybrid^30−32^ and double hybrid (DH) functionals^33−35^ and successively it has been directly employed to construct high-level XC functionals based on AC models (ACM) interpolating between known limits of the AC integrand.^36−43^ Recently, it has also been employed in the context of the Hartree–Fock (HF) theory^44,45^ to develop corrections to the Møller–Plesset perturbation series.^46^

The XC functionals based on ACMs have the general form
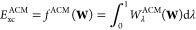
1where , with *W*_0_ = *E*_x_ being the exact exchange energy, *W*_0_^′^ = 2*E*_c_^GL2^ being twice the GL2 correlation energy,^11^ and *W*_∞_ and *W*_∞_^′^ being the indirect part of the minimum expectation value of the electron–electron repulsion for a given density and the potential energy of coupled zero-point oscillations around this minimum, respectively.^39,47^ The model *W*_λ_^ACM^ is designed to mimic the exact but unknown *W*_λ_, in particular by considering the known asymptotic expansions^11,39,40,47^
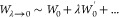
2
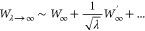
3

In recent years, several ACMs have been tested for various chemical applications showing promising results,^48,49^ especially in the description of non-covalent interactions.^46,50^ However, most of these recent studies have been performed within the HF–AC framework, that is, as post-HF calculations. Conversely, little attention has been devoted to DFT-based ACM functionals. The main reason for this is that in the HF case, the ACM is applied on top of the HF ground state,^44,45^ which is a simple and well defined reference; on the contrary, in the DFT framework, the ACM-based XC functional should be in principle applied inside the KS equations in a self-consistent-field (SCF) fashion. This requirement is not trivial because ACM-based functionals are in general not simple explicit functionals of the density but are instead complicated expressions depending on KS orbitals and orbital energies as well (through *E*_x_ and *E*_c_^GL2^). One notable exception are the MCY functionals^51^ which use semilocal approximations to set the interpolation points along the AC integrand, thus allowing for a relatively straightforward SCF implementation. In the most general case considered in this work, however, ACM functionals are fifth rung functionals and thus, in practice, also in the context of DFT, they are always applied in a post-SCF scheme using precomputed DFT densities and orbitals.^48,52^ In this way, the results depend significantly on the choice of the reference density and orbitals, making the whole method not fully reliable.^48^ On the other hand, an exploratory study of the XC potential derived from ACM models has shown that this possesses promising features, indicating that SCF calculations with ACM-based functionals might be an interesting path to explore.^53^

In this work, we tackle this issue by introducing an SCF implementation of the ACM potential and applying it to some test problems in order to verify its ability to describe different properties and systems. One important aim of this work is in fact to measure and assess the capabilities of some of the most popular ACM presently available in literature. To this purpose the use of a proper SCF procedure is crucial as the level of accuracy of such methods can be inspected independently of an arbitrary reference ground-state as in previous works. In fact, for any density functional, the energy error can be decomposed into a contribution due to the approximate nature of the functional (intrinsic error) and that due to the approximate density used in the calculation (relaxation error).^54,55^ When the functional is evaluated on an arbitrary (non-SCF) density, the relaxation error may become important and the whole performance can be influenced by the choice of the density. Indeed, recent studies have shown how this effect can be used to improve DFT results by choosing accurate non-SCF densities.^55,56^ Nevertheless, within this framework, it is difficult to really understand the accuracy of the functional form itself and therefore to plan new advances. On the other hand, the use of a proper SCF procedure provides a well-defined reference for assessing the intrinsic errors. This is an extremely important point to clarify in view of future ACM developments. Note that such a development of new and possibly more accurate ACMs will instead not be covered in this work but left to upcoming publications. The development work performed here will instead focus on a second important goal aimed at solving some open problems with the ACM potential that hinder its straightforward SCF implementation. These problems originate mainly from the naive treatment used so far for the large-λ contributions *W*_∞_ and *W*_∞_^′^ which causes an unphysical behavior in the ACM potential. Hence, in this article, we develop new approximations for both *W*_∞_ and *W*_∞_^′^ that preserve the accuracy for energies and remove the limitations on the potential side. As a byproduct of this work, we obtain useful strong-correlation generalized gradient approximations that prove to be very robust for the description of the Harmonium atom and the H_2_ dissociation.

In the following, we present the theory behind SCF implementation of ACM functionals and the construction of new *W*_∞_ and *W*_∞_^′^ approximations. Afterward, we present some interesting preliminary results obtained for model and real systems.

## Theory

To perform SCF ACM calculations we need to deal with the potential arising from the functional derivative of the energy of eq 1, that is^53^
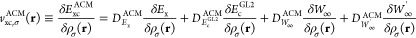
4where *D*_*j*_ = ∂*f*^ACM^/∂*j* with *j* = *E*_x_, *E*_c_^GL2^, *W*_∞_, *W*_∞_^′^. As discussed in ref (53), the potential in eq 4 requires a combination of OEP (for *E*_x_ and *E*_c_^GL2^) and generalized gradient approximation (GGA) approaches (for *W*_∞_ and *W*_∞_^′^). Thus, it resembles the OEP–SCF implementation of the DH functionals reported in refs (57) and (58). In more details, the  and  functional derivatives are obtained by solving the OEP equation which reads^9,10,12,59−61^
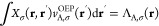
5with A = X, C denoting the exchange and correlation parts, respectively. The inhomogeneity on the right hand side of eq 5 is given by
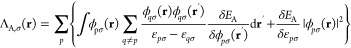
6and the static KS linear response function is
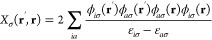
7

All quantities are evaluated using orbitals ϕ_*p*σ_ and eigenvalues ε_*p*σ_ in a given cycle of KS SCF procedure (further details can be found in refs (17), (57), (58), (62), and (63)). We note, however, that there is a significant difference between ACM and DH approaches: in the former, the coefficients  and  are not fixed empirical parameters as in DH, but are well-defined (non-linear) functions of *E*_x_, *E*_c_^GL2^, *W*_∞_, *W*_∞_^′^.^53^

### Approximations for the Strong-Interaction Limit

Another important issue to consider in the SCF implementation of the ACMs is related to the treatment of *W*_∞_ and *W*_∞_^′^, which describe the λ → ∞ limit of the AC integrand. It can be proven that both *W*_∞_ and *W*_∞_^′^ display a highly non-local density dependence.^64−68^ This is accurately described by the strictly correlated electron (SCE) formalism,^39,47^ which is however computationally very demanding and nontrivial to evaluate. Therefore, the λ → ∞ limit is usually approximated by simple semilocal gradient expansions (GEA) derived within the point-charge-plus-continuum (PC) model^38^
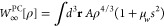
8
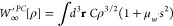
9where  is the reduced gradient of the density, *A* = −9(4π/3)^1/3^/10, *C* = 1/2(3π)^1/2^, μ_*w*_ = −3^1/3^(2π)^2/3^/35 ≈ −0.1403, and μ_*w*′_ = −0.7222 (slightly different estimates are possible for μ_*w*′_, see, e.g., refs (36) and (39)). The GEAs of eqs 8 and 9 yield, at least for small atoms, energies that are quite close to the accurate SCE values. However, when *s* is large, for example, in the tail of an exponentially decaying density, they fail, giving functional derivatives that diverge.^53^ This is a severe drawback that does not allow these approximations to be used directly in an SCF implementation.

To remedy this limitation we consider here a simple GGA approximation, named, harmonium PC (hPC) model, based on the Perdew–Burke–Ernzerhof (PBE) exchange enhancement factor^69^ that recovers the GEAs of eqs 8 and 9 in the slowly varying regime, is well behaved everywhere, and reproduces as close as possible the SCE values for both *W*_∞_ and *W*_∞_^′^. Thus, we have
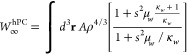
10
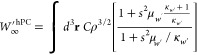
11where κ_*W*_ = −7.11 and κ_*W*′_ = −99.11 have been fixed such that *W*_∞_^hPC^ and *W*_∞_^′hPC^ recover exactly the corresponding SCE values for the harmonium atom at ω = 0.5:^70^ with this value, the degree of correlation resembles that of the He atom and a simple analytical density is obtained. We note that a previous attempt to develop GGAs for *W*_∞_ and *W*_∞_^′^, the modified PC (mPC) model of ref (71), yields results that are quite far from both the PC and the SCE values, in particular *W*_∞_^′^ does not even recover the PC model in the small *s* limit. In fact, the mPC GGAs have been derived for the quasi-two-dimensional density regime^71^ and their application in three-dimensional systems, for example for the total correlation of atoms, is highly based on an error cancellation between the quite inaccurate values of *W*_∞_ and *W*_∞_^′^.^71^ In particular,  has been designed to compensate the inaccuracies of *W*_*∞*_^mPC^ for the ISI functional, but this error compensation cannot work for other ACMs (especially those, as SPL, using only *W*_∞_).

**Figure 1:**
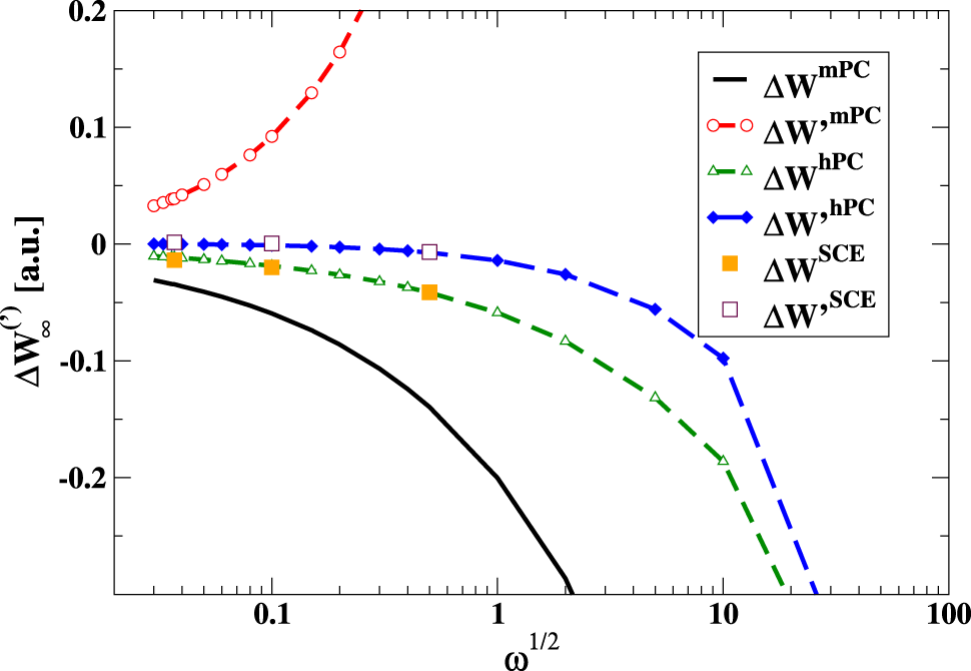
Differences between the values of *W*_∞_ and *W*_∞_^′^ computed with hPC and mPC formulas and the corresponding *W*_∞_^PC^ and *W*_∞_^′PC^ data (Δ*W*_∞_^method^ = *W*_∞_^method^ – *W*_∞_^PC^; Δ*W*_∞_^′method^ = *W*_∞_^′method^ – *W*_∞_^′PC^) for the harmonium atom at various values of the confinement strength ω. For reference, some available accurate SCE values are also reported.^70^

To understand the performances of the different approximations for the strong-interaction functionals, we report in Figure 1 the differences between the values of *W*_∞_ and *W*_∞_^′^ computed with the two GGAs and the PC model, for the Hooke atom at different confinement strengths ω. The corresponding values for those instances of ω for which exact SCE reference data are available are also reported in Table 1.

**Table 1:** *W*_∞_ and *W*_∞_^′^ Energies (in Ha) for Three Values of ω for Which Hooke’s Atom has Analytical Solutions^72^ and Exact SCE Reference Data Are Available^70^^a^

ω	SCE	PC	hPC	mPC
*W*_∞_				
0.0365373	–0.170	–0.156	–0.167	–0.191
0.1	–0.304	–0.284	–0.303	–0.344
0.5	–0.743	–0.702	–0.743	–0.841
MARE		6.78%	0.70%	12.90%
*W*_∞_^′^				
0.0365373	0.022	0.021	0.021	0.060
0.1	0.054	0.054	0.053	0.146
0.5	0.208	0.215	0.208	0.562
MARE		2.64%	2.13%	171.10%

a Hooke’s atom is usually considered to be in the strong correlation regime when the density displays a maximum away from the center of the harmonic trap, which happens^73^ for ω ≲ 0.0401. The last line of each panel reports the MARE.

**Figure 2:**
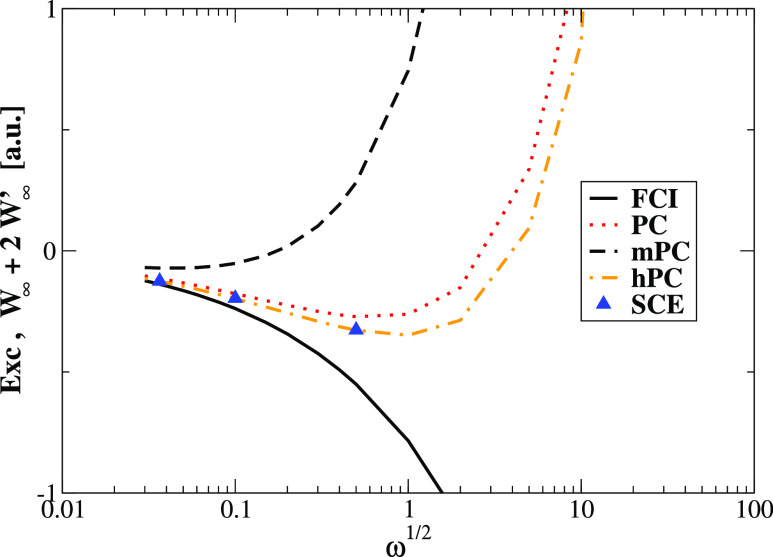
Comparison of the leading term of the XC energy  in the strong interacting regime of the Hooke’s atom calculated using different models with FCI data.^52^

We see that, unlike mPC, the hPC model reproduces very well both the *W*_∞_ and *W*_∞_^′^ accurate SCE values,^70^ being comparable to and even superior to the original PC model. This performance is not trivial because hPC was parameterized only on a single instance of the Hooke’s atom (ω = 0.5) but turns out to be very accurate for the whole range of confinement strengths. In particular, Figure 2 shows that in the small ω range (strong interaction limit of the Hooke’s atom) hPC yields the best estimation of the XC energy *E*_xc_ = *W*_∞_ + 2*W*_∞_^′^, being slightly better than PC, while the mPC method fails completely.

**Figure 3:**
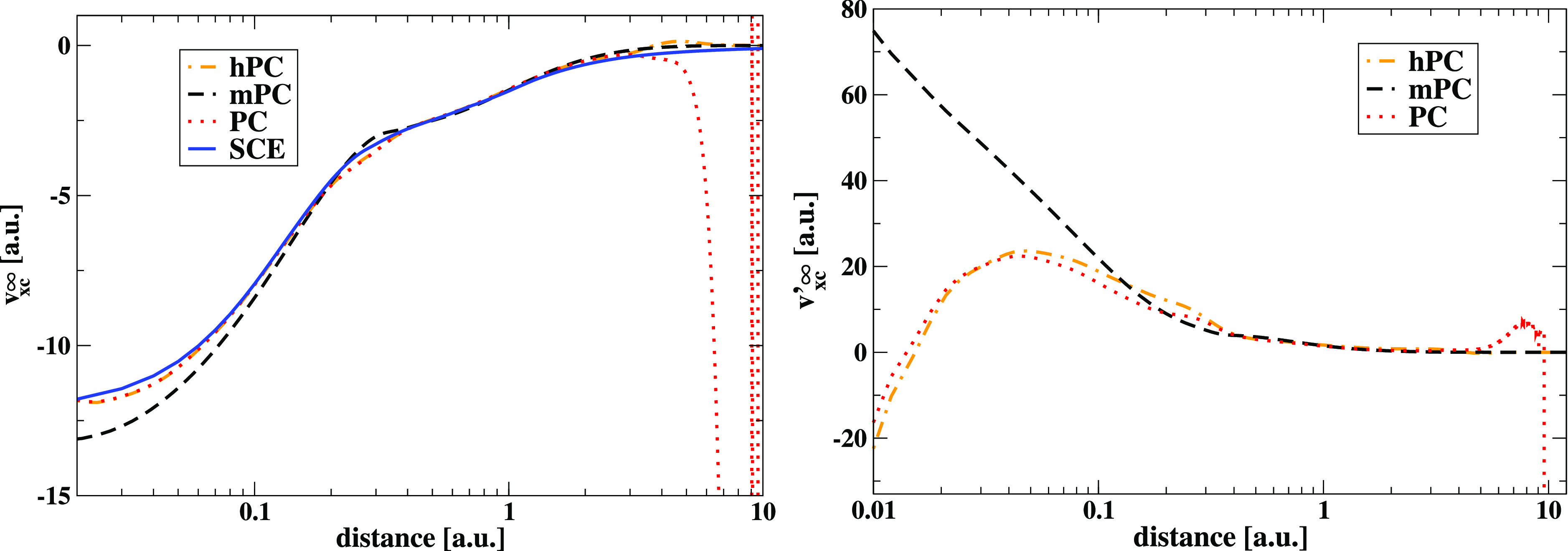
Comparison between (a) *v*_xc_^∞^(**r**) = δ*W*_∞_/δρ(**r**) and (b) *v*_xc_^′∞^(**r**) = δ*W*_∞_^′^/δρ(**r**) potentials computed from different models for the Ne atom (using EXX densities).

An additional assessment is provided in Table 2 and Figure 3 where real atoms are considered both for SCE energies and SCE potentials. Also in this case the results of the hPC functional are in line with or better than the PC model, that was originally parametrized against the He atom, indicating once more the robustness of the hPC method. As anticipated, the mPC is instead quite far from the reference, especially for *W*_∞_^′^.

**Table 2:** Values of *W*_∞_ and *W*_∞_^′^ for the He, Be, and Ne Atoms Obtained from Different Models and Using EXX Densities; We Use Atomic Units^a^

	SCE	PC	hPC	mPC
*W*_∞_				
H	–0.3125	**–0.3128**	–0.3293	–0.4000
He	–1.500	–1.463	**–1.492**	–1.671
Be	–4.021	–3.943	**–3.976**	–4.380
Ne	–20.035	**–20.018**	–20.079	–21.022
MARE		**1.15%**	1.81%	13.31%
*W*_∞_^′^				
H	0	0.0426	**0.0255**	0.2918
He	0.621	0.729	**0.646**	1.728
Be	2.59	2.919	**2.600**	6.167
Ne	22	24.425	**23.045**	38.644
MARE		13.71%	**3.05%**	130.67%

a The results which agree best with SCE values^39,47^ are highlighted in bold. The last line of each panel reports the MARE [for *W*_∞_^′^ the H results are excluded]. The *W*_∞_^′SCE^ reference data are reported with the same precision of as in ref (39).

## Computation Details

All calculations have been performed with a locally modified ACESII^74^ software package. As in our previous studies,^17,53,57,58,62,63,75,76^ in order to solve OEP equations, we have employed the finite-basis set procedure of refs (77) and (78). In calculations, we employed the basis sets detailed below and tight convergence criteria (SCF: 10^–8^). In general, the convergence criteria were met within several cycles of the SCF procedure.

In order to solve algebraic OEP equations, the truncated singular-value decomposition (TSVD) of the response matrix was employed. The cutoff criteria in the TSVD procedure were set to 10^–6^. For technical details on this type of calculations, we refer the reader to refs (17) and (63).

As reference data, we have considered the coupled-cluster single double and perturbative triple [CCSD(T)]^79^ results obtained in the same basis set in order to make a comparison on the same footing and to reduce basis set related errors. In particular, we have considered a comparison with CCSD(T) relaxed densities, the corresponding KS potentials obtained via KS inversion,^80^ and the total CCSD(T) energies. In the assessment, we have considered several properties, that is:

- **total energies:** the total energies have been calculated for the systems listed in Table 1 in ref (63) using an identical computational setup as in the same paper. A summary of the employed basis sets is also reported in the Supporting Information. We remark that, although total energies are not very important in practical chemical applications, they are important observables and are especially useful as indicators of the quality of the ACM interpolation.
- **Dipole moments:** for selected systems (H_2_O, HF, HCl, H_2_S, and CO), we have calculated the dipole moments using SCF densities for various methods. This is a direct test of the quality of self-consistent densities obtained within all approaches. The uncontracted aug-cc-pVTZ basis set of Dunning^81^ was used for all systems together with geometries taken from ref (82).
- **HOMO and HOMO–LUMO gap energies:** as in refs (63) and (83), we have computed the HOMO and HOMO–LUMO gaps, respectively, for the same set of systems as in the case of total energies. In the case of HOMO energies, the reference data have been taken from ref (83), whereas the HOMO–LUMO gap energies have been obtained from applying the KS inversion method^80^ taking as a starting point the CCSD(T) relaxed density matrix as in ref (63).
- **correlation potentials and densities:** as in our previous studies,^17,63,84,85^ here we also investigate the quality of correlation potentials and densities^17,86,87^ looking at their spatial behavior. Both quantities are obtained from fully SCF calculations. The densities are analyzed in terms of correlation densities defined as Δρ_c_ = ρ^method^ – ρ^X^, where ρ^X^ is the density obtained from the exact exchange only (X = EXX)^60^ or Hartree-Fock (HF) (X = HF) calculations for DFT and WFT methods, respectively. The Ne atom OEP calculations have been performed in a fully uncontracted ROOS-ATZP^88^ basis set, whereas for the CO molecule, the uncontracted cc-pVTZ^89^ basis sets were employed.
- **dissociation of H**_**2**_**:** fully self-consistent and post-SCF calculations, using OEP EXX orbitals, have been performed in the spin restricted formalism using the uncontracted aug-cc-pVTZ basis set. For comparison, PBE, MP2, GL2@EXX, and FCI data are also reported.
- **correlation energies of Hooke’s atoms:** as previously,^52,90,91^ we have performed the calculation for various values of ω in the Hooke’s atom model^92^ ranging between 0.03 (strong interaction) and 1000 (weak interaction) using a even-tempered Gaussian basis set from ref (93). For comparison, the ACM correlation energies have been calculated at both @EXX and @SCF reference orbitals.

## Results

We have performed a series of SCF ACM calculations to investigate the performance of these methods in the KS framework. In particular, we have considered the interaction–strength–interpolation (ISI)^36^ and Seidl–Perdew–Levy (SPL)^40^ ACMs. Unless explicitly stated, the hPC model has been used to describe the strong-interaction limit in all calculations. Moreover the bare GL2 (for SCF calculations OEP-GL2^12^) approach is also reported. The ISI model for *W*_λ_ has in general a larger deviation from linearity than SPL (which does not depend on *W*_∞_^′^ too), whereas GL2 corresponds to the linear approximation *W*_λ_ = 2*E*_GL2_ λ. Thus, the comparison of ISI with SPL and GL2 gives information on the importance of the shape of the ACM interpolation form.

In Table 3, we show the total energies computed with the various methods for a test set of 16 closed-shell atoms and small molecules, namely, He, Be, Ne, Mg, Ar, HF, CO, H_2_O, H_2_, He_2_, Cl_2_, N_2_, Ne_2_, HCl, NH_3_, and C_2_H_6_.

**Table 3:** Total Energies (Ha) Calculated with Different Methods Self-Consistently (@SCF) or on top of EXX Orbitals (@EXX), for Several Functionals^a^

	@SCF			@EXX			
system	ISI	SPL	GL2	ISI	SPL	GL2	CCSD(T)
He	–2.90089	–2.90043	–2.90780	–2.90191	–2.90148	–2.90925	–2.90253
Be	–14.67318	–14.67551	not. conv.	–14.67102	–14.67278	–14.69013	–14.66234
Ne	–128.93274	–128.94313	–128.98863	–128.92733	–128.93628	–128.97770	–128.89996
Mg	–199.86915	–199.86937	–199.88275	–199.86560	–199.86569	–199.87826	–199.82815
Ar	–527.51661	–527.53309	–527.58461	–527.51478	–527.53095	–527.58181	–527.45748
H_2_	–1.17039	–1.16972	–1.18107	–1.17019	–1.16953	–1.18060	–1.17273
He_2_	–5.80177	–5.80086	–5.81560	–5.80167	–5.80075	–5.81539	–5.80506
N_2_	–109.58263	–109.61715	–109.75090	–109.56105	–109.58609	–109.68725	–109.47628
Ne_2_	–257.86564	–257.88644	–257.97751	–257.85475	–257.87266	–257.95552	–257.80003
HF	–100.43787	–100.45019	–100.50368	–100.43148	–100.44188	–100.48965	–100.39579
CO	–113.35397	–113.38496	–113.51191	–113.32766	–113.34760	–113.43484	–113.25738
H_2_O	–76.42686	–76.44091	–76.50285	–76.42076	–76.43270	–76.48790	–76.38692
HCl	–460.58531	–460.58876	–460.61411	–460.58227	–460.58550	–460.61020	–460.50933
Cl_2_	–919.93674	–919.94378	–919.99349	–919.92413	–919.93022	–919.97763	–919.77032
NH_3_	–56.55283	–56.56435	–56.62446	–56.54859	–56.55876	–56.61412	–56.52332
C_2_H_6_	–79.80517	–79.82045	–79.92279	–79.79876	–79.81239	–79.90830	–79.76414
ME	–50.00	–61.08^b^	–120.85	–43.14	–52.09	–99.17	
MAE	50.91	62.25^b^	120.85	43.96	53.16	99.17	
MARE	0.055%	0.071%^b^	0.162%	0.048%	0.062%	0.150%	

a CCSD(T) results are given as a reference. The last rows report the mean error (ME, in mHA), MAE (in mHA), and the MARE (in percent). For OEP–GL2, all the averages exclude the Be atom that for this functional has not converged. Not. conv.—not converged.

b Without Be.

We see that ISI@SCF and SPL@SCF perform quite well, giving errors roughly half that of OEP–GL2. For comparison, we acknowledge that the PBE functional^69^ yields a mean absolute relative error (MARE) of 0.11%, which is twice as large as that of ISI@SCF.

Nevertheless, we have to acknowledge that the performance has further margins of improvement. For example the MAEs of MP2 and OEP2-sc (not reported) for the same test are 20 and 17 mHa, respectively. We can trace back most of this difference to the fact that the use of KS eigenvalues, as in ISI, SPL, and OEP–GL2, requires a quite large AC curvature (i.e., second derivative with respect to λ) to yield accurate results, whereas this is not the case for MP2 and OEP2-sc that employ HF-quality eigenvalues. Then, KS-based methods need much more accurate ACMs to compete with HF-based ones. This is also confirmed observing that in Table 3, ISI is generally better than SPL, as the former is a more advanced ACM than the latter.

A second, related observation is that the ISI and SPL results suffer from a small relaxation error that worsens slightly the performance (with respect to using EXX orbitals). This effect might be related to the fact that the considered ACMs were developed in the context of post-SCF calculations and, as a result, may include some inherent error cancellation which is lost when they are evaluated using a (more accurate) SCF density. To better understand this trend, we define the quantity
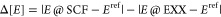
12which considers the absolute error difference [with respect the reference, i.e., CCSD(T)] going from EXX orbitals to SCF orbitals (a negative value means that SCF orbitals give better accuracy than EXX orbitals). The values of Δ[*E*] for ISI, SPL, and OEP–GL2/GL2 are 7.0, 9.1, and 16.9 mHa, respectively. Despite the Δ[*E*] values all being positive (i.e., calculations using EXX orbitals are more accurate) they decrease going from GL2 to SPL and then from SPL to ISI, showing again that increasing the complexity/accuracy of the ACM can yield better SCF potentials and relaxed total energies.

**Figure 4:**
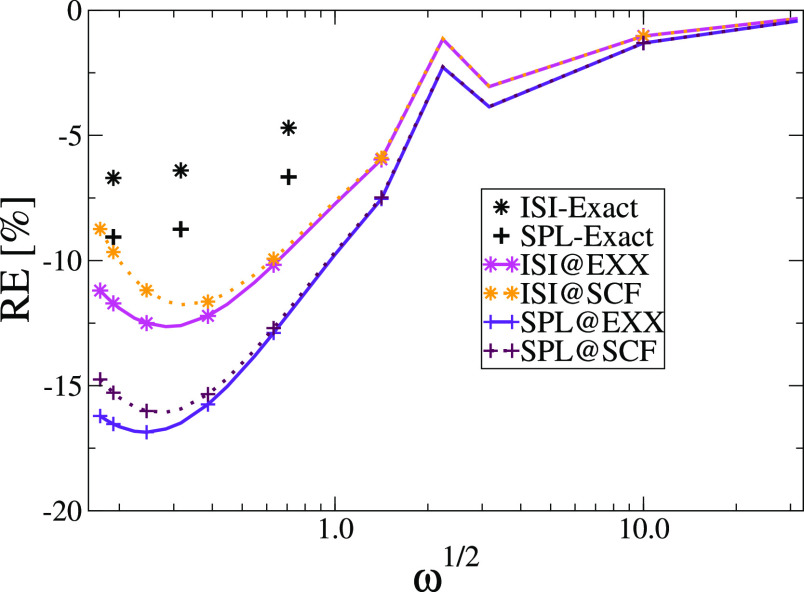
Relative error on correlation energies of harmonium atoms for various values of ω computed at @SCF and @EXX orbitals for ISI and SPL functionals using the hPC model for the strong-interaction functionals. The errors have been computed with respect to FCI data obtained in the same basis set.^93^ The exact ISI and SPL values are taken from ref (70) and are obtained by inserting exact densities into the ISI and SPL functionals, including the exact treatment (SCE) of the strong-interaction limit.

Interestingly, an opposite effect of the density relaxation is found in the harmonium atom, as shown in Figure 4, where if we look at small values of the confinement strength, where the relaxation becomes more important, the SCF results are better with respect to the ones obtained using EXX orbitals and density (for both ISI and SPL). This depends on the fact that at these regimes, the true density is very different from the EXX one, and thus, the SCF procedure produces a significant improvement on the density. This also traces back to the use of hPC which yields accurate strong-correlation potentials; we note in fact that the accuracy of both ACMs with the hPC model is very high (compare, e.g., with Figure 3 of ref (52)). Conversely using the mPC model only ISI results are rather accurate because of error compensation effects between the *W*_∞_^mPC^ and the  terms, while SPL ones, where only *W*_∞_^mPC^ is used, are rather poor (see Figure S2 in the Supporting Information). This is an important indication of the importance of using proper strong-correlation approximations, delivering both good energies and potentials.

In Table 4, we report the dipole moments of some selected systems, from the SCF density. The results for CO are reported separately because they are qualitatively different and deserve a distinct analysis.

**Table 4:** Dipole Moments (in Debye) for Some Selected Systems Calculated Using Self-Consistent Densities^a^

					MAE		
method	H_2_O	HF	HCl	H_2_S	CCSD(T)	exp.	CO
OEPx	2.043	1.954	1.279	1.171	0.121	0.180	–0.265
GL2	1.616	1.531	1.061	1.004	0.187	0.145	1.703
SPL	1.758	1.654	1.085	1.024	0.110	0.080	0.940
ISI	1.809	1.699	1.093	1.030	0.083	0.060	0.692
OEP2-sc	1.885	1.786	1.185	1.094	0.018	0.073	0.355
CCSD(T)	1.904	1.809	1.170	1.079		0.065	0.153
Exp.	1.855	1.820	1.080	0.970			0.122

a Experimental data are taken from ref (82). The MAE of H_2_O, HF, HCl, and H_2_S with respect to CCSD(T) and experimental results is also reported.

For H_2_O, HF, HCl, and H_2_S, a comparison with the CCSD(T) data shows that ISI is quite effective in predicting the dipole moments being slightly better than SPL and twice as good as GL2 [for comparison PBE gives in this case a mean absolute error (MAE) of 0.092 Debye with respect CCSD(T)]. Anyway, as already observed for the total energies, there are important margins of improvement as testified by the OEP2-sc performance that is definitely better than the ISI one. As already discussed, we can trace back the limitations of ISI and SPL not only in part to relaxation effects but also on the fact that, working in a pure SCF KS framework, it is very hard for the ACM to provide a proper curvature of the AC integrand curve as to get accurate KS orbital energies; consequently, the orbital-dependent energies are also negatively affected. For the case of CO, these effects are even more evident. In this case, in fact, OEPx predicts a qualitatively wrong dipole moment but GL2 largely over-corrects it, indicating that the linear behavior of the AC integrand needs to be significantly improved. Both ISI and SPL can partially achieve this task, halving the error with respect to GL2, but still they yield quite overestimated dipole moments.

As a next step, we consider in Table 5 the highest occupied molecular orbital (HOMO)–lowest unoccupied molecular orbital (LUMO) gaps obtained from different methods. As it could be expected both ACMs correct the general overestimation of gaps given by the OEPx but in doing so, they overestimate the correlation effects yielding gaps that are too small in most cases. Thus, we obtain MAEs of 0.68 and 0.52 eV for SPL and ISI, respectively, to be compared with the OEP2-sc MAE of 0.21 eV. We note anyway that the ISI and SPL results are clearly better than conventional semilocal functionals (PBE gives a MAE of 0.97 eV). Moreover, we note that by improving the quality of the ACM (GE2 → SPL → ISI) the description of the HOMO–LUMO gap is also significantly improved. Similar considerations apply as well for the HOMO energies (see Table 6). At the ISI level, the HOMO is shifted to higher energy with the almost the same MARE as OEPx (which is shifted to lower energy). Again, the ISI approach is better than SPL and much better than GL2 (as well as PBE with a MARE of 38.3%).

**Table 5:** HOMO–LUMO Energy Gap (eV) for Different Systems as Obtained from Several Methods^a^

	@SCF					
system	OEPx	GL2	OEP2-sc	SPL	ISI	KS[CCSD(T)]
He	21.60	20.95	21.32	21.23	21.23	21.21
Be	3.57	not. conv.	3.63	3.40	3.47	3.61
Ne	18.48	14.12	16.45	15.17	15.60	17.00
Mg	3.18	3.40	3.33	3.38	3.38	3.36
Ar	11.80	10.95	11.43	11.08	11.17	11.51
H_2_	12.09	12.03	12.13	12.12	12.12	12.14
He_2_	21.28	20.64	21.02	20.81	20.81	20.56
N_2_	9.21	6.73	8.37	7.68	7.99	8.55
Ne_2_	17.84	13.49	15.75	14.41	14.83	16.23
HF	11.36	7.80	9.84	8.70	9.08	10.30
CO	7.77	5.87	7.22	6.68	6.90	7.29
H_2_O	8.44	5.99	7.49	6.73	7.03	7.75
HCl	7.82	7.10	7.52	7.11	7.14	7.55
Cl_2_	3.90	2.65	3.35	2.74	2.78	3.29
NH_3_	6.97	5.30	6.35	5.78	5.98	6.54
C_2_H_6_	9.21	8.24	8.85	8.51	8.62	8.95
ME	+0.54	–1.13^b^	–0.11	–0.64	–0.48	
MAE	0.52	1.15^b^	0.21	0.68	0.52	
MARE	6.49%	12.16%^b^	1.86%	7.49%	5.71%	

a The last column reports the reference CCSD(T) data obtained from inverse method. The last lines report the MAE, and the MARE with respect to the CCSD(T) results. Not. conv.—not converged.

b without Be.

**Table 6:** HOMO Orbital Energies (eV) for Different Systems as Obtained from Several Approaches^a^

	@SCF					
system	OEPx	GL2	OEP2-sc	SPL	ISI	CCSD(T)
He	–24.98	–24.23	–24.55	–24.46	–24.39	–24.48
Be	–8.41	not. conv.	–8.89	–9.47	–9.32	–9.31
Ne	–23.38	–17.66	–20.14	–18.98	–19.48	–21.47
Mg	–6.88	–8.04	–7.33	–7.93	–7.91	–7.57
Ar	–16.08	–14.94	–15.34	–15.11	–15.20	–15.63
H_2_	–16.17	–16.34	–16.30	–16.25	–16.13	–16.41
He_2_	–24.92	–24.14	–24.47	–24.38	–24.30	–24.48
N_2_	–17.17	–11.32	–15.65	–13.09	–13.78	–15.51
Ne_2_	–23.05	–17.45	–19.98	–18.80	–19.31	–21.34
HF	–17.48	–12.16	–14.57	–13.52	–14.03	–15.96
CO	–15.02	–10.64	–13.21	–12.18	–12.70	–13.94
H_2_O	–13.69	–9.01	–11.27	–10.39	–10.87	–12.50
HCl	–12.92	–11.94	–12.28	–12.04	–12.08	–12.59
Cl_2_	–12.06	–9.92	–10.85	–10.14	–10.22	–11.45
NH_3_	–11.56	–8.37	–9.91	–9.34	–9.65	–10.78
C_2_H_6_	–13.21	–11.39	–12.20	–11.93	–12.07	–13.01
ME	–0.65	+1.97^b^	+0.59	+1.15	+0.93	
MAE	0.89	2.49^b^	0.62	1.22	0.98	
MARE	6.12%	13.68%^b^	4.36%	8.28%	6.67%	

a In the last column, we report reference HOMO energies from ref (62). The last lines report the MAE, and the MARE calculated with respect to the CCSD(T) results. Not. conv.—not converged.

b Without be.

**Figure 5:**
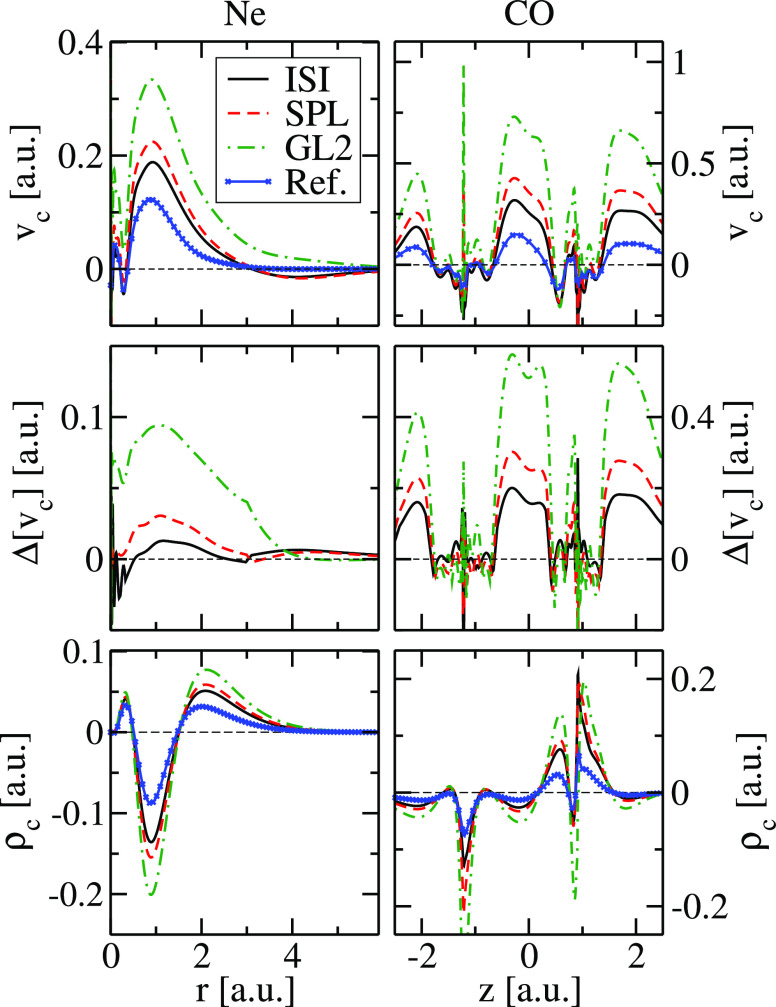
Correlation potentials (top panels), Δ[*v*_c_] (middle), and correlation density (bottom) for the Neon atom (left) and CO molecule (right) obtained using several ACM–SCF methods. Reference means the CCSD(T) data using the method from ref (80).

Then, we consider the correlation potentials for two typical systems, the Ne atom and the CO molecule. In the top panels of Figure 5, we see that the ACMs provide a quite good description of the correlation potential for the two systems, improving significantly over GL2. Nevertheless, with respect to reference data there are still some limitations, for example, a moderate overestimation of the correlation potential in valence regions. This characteristic corresponds to an overestimation of shell oscillations in the SCF density, as indicated in the bottom panels of Figure 5, where we report the correlation density ρ_c_, that is, the difference between the density obtained with a correlated method and its exchange-only version.

In the central panels of Figure 5, we report the values Δ[*v*_c_(**r**)], which is defined, in analogy to eq 12 as
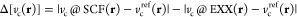
13

These show, point-by-point whether or not the SCF procedure improves the correlation potential with respect to EXX orbitals. As we found for energies, the SCF correlation potentials are less accurate, but the error reduces with more accurate ACM functionals. This feature is also evident for the correlation density, see bottom panels. In this context, we should however also point out that the ACM-SCF density does not correspond to the exact linear response density.^94−96^

**Figure 6:**
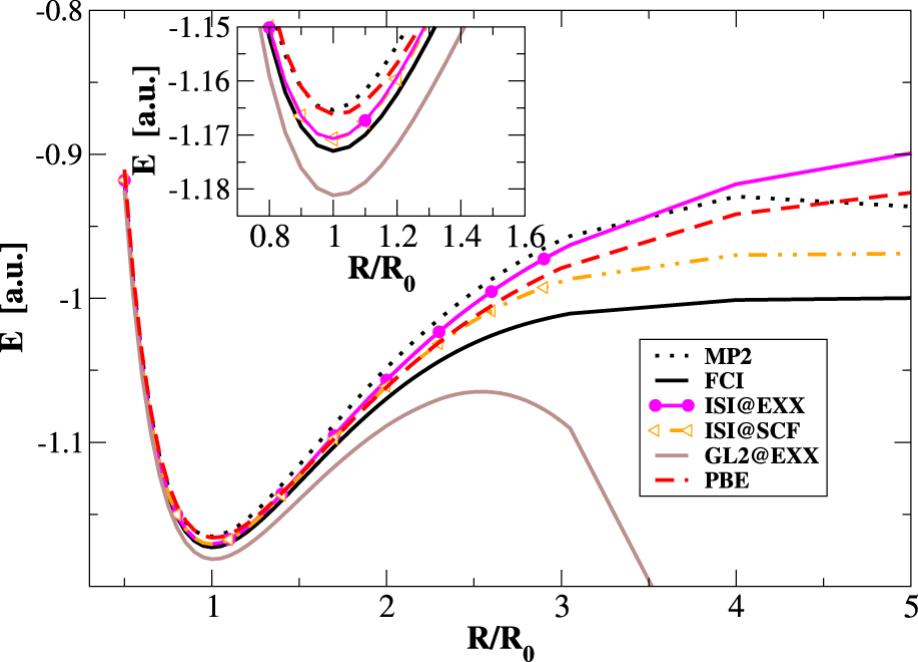
Total energy of the H_2_ molecule as it is stretched calculated with the various methods. The inset presents the same data around the equilibrium distance.

As a final case, we consider in Figure 6 the potential energy surface for the dissociation of the H_2_ molecule, in a restricted formalism,^97^ which is one of the main DFT challenges,^97,98^ and was previously investigated in the ACM framework.^43,99,100^ While both MP2 and GL2@EXX diverge at large distances, ISI@SCF nicely reproduces the exact FCI curve, much better than ISI@EXX, see also ref (48). Thus, the SCF procedure turns out to be quite important showing that, despite some limitations discussed above, it is crucial to include important correlation effects into the orbitals. For SPL (see Figure S1 in the Supporting Information), similar trends are found; the SPL@SCF curve for *R*/*R*_0_ > 2.5 first increases and then decreases asymptotically, a behavior which is clearly incorrect and depends on some drawbacks of the SPL functional to describe the limit for large distances, which is more influenced by the strong correlation.

The limit for very large distances, well beyond *R*/*R*_0_ > 5, is numerically tricky, but it can be computed exactly using the hydrogen atom with fractional spins, H(1/2,1/2), that is, with half spin up and half spin down.^101^ For this system, we have *E*_GL2_ → −∞ so that the ISI XC energy reduces to^36^
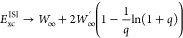
14with . The potential is thus a simple linear combination of the EXX potential and the GGA potential from *W*_∞_ and *W*_∞_^′^. For the SPL approach, we have simply that *E*_xc_^SPL^ → *W*_∞_ and thus the potential is just δ*W*_∞_/δρ(**r**).

The errors for different methods and orbitals are reported in Table 7.

**Table 7:** Total Energy Error for H(1/2,1/2) in kcal/mol for Different Methods and Orbitals, Using a Geometric Series Basis-Set with 17 Uncontracted Gaussian Functions, 10^4^ as the Maximum Exponent, and 2.5 as the Geometric Progression Factor^a^

	@EXACT	@SCF	IDD
PBE	54.7	51.5	0.103
EXX	196.1	178.6	0.260
SPL-PC	**–0.4**		
SPL-mPC	–109.8	–114.9	0.125
SPL-hPC	**–21.0**	**–21.4**	**0.024**
ISI-PC	27.4		
ISI-mPC	90.2	83.7	0.151
ISI-hPC	23.6	**19.4**	**0.107**

a The last column reports the IDD error, that is, ∫d*r*4π*r*^2^|ρ(*r*) – ρ^exact^(*r*)|. Note that self-consistent PC calculations do not converge. The best two ACM results are reported in boldface.

At the exact density (ρ(*r*) = exp(−2*r*)/π) SPL–PC gives an extremely accurate total energy but the same method fails for the SCF calculation. The SPL-mPC approach strongly underestimates the total energy, while the SPL-hPC gives a much lower error, both for the exact and the SCF densities. At the ISI level, all the energies are higher and the ISI-hPC@SCF is the most accurate approach. Note, however, that ISI-PC can be made exact with a proper choice of the parameters.^38^ Note also that EXX fails for this system and PBE is also quite inaccurate.

When SCF effects are considered, PBE, EXX, ISI-mPC, and ISI-hPC yield a slight improvement with respect to the case when the exact density is used. Because the integrated density difference (IDD) is not zero in all cases, this is a clear signature that all methods display some error compensation effect. Moreover, some methods give important convergence issues: the simple PC model does not converge, as explained above; the mPC model converges but the errors are very large, about twice the PBE ones. Instead, the ISI-hPC is very good for both the considered densities, having the best accuracy among all functionals and performing even better than all the functionals considered in Table 5 of ref (97). Note that the good accuracy of the ISI-hPC with respect to ISI-mPC is not related to the previously mentioned error cancellation between an incorrect SCF density and an incorrect energy. In fact, the IDD error is significantly smaller going from ISI-hPC to ISI-mPC. Interestingly, the same arguments hold when comparing SPL-hPC to SPL-mPC, thus confirming the high quality of the hPC functional. Note that the almost vanishing IDD value for the SPL-hPC approach is a particular case, and all methods with IDD ≲ 0.1 show a quite accurate density. The accuracy of the ISI-hPC@SCF approach for the H_2_ dissociation limit is thus quite significant, considering that it uses full exact exchange and a combination of GL2 and a GGA functional without empirical parameters, in contrast to other approaches that use more complex constructions or extensive fitting on molecular data.^98,102^

## Conclusions

In this paper, we have shown that it is possible to use ACM-based XC functionals in a full SCF procedure. This solves a long-standing issue in DFT as all the previous calculations with ACM functionals had been done in a post-SCF fashion using GGA or EXX orbitals. This opens the way to new applications and even basic studies in this context, removing the need for a post-SCF procedure and all the related sources of inaccuracy. Of course, despite the ACM–SCF procedure presented here is well defined, conceptually clean and fully capable of producing important results, is it fair to state that the whole method is not yet optimized and straightforward to apply especially because it is strictly related to the OEP approach used for the treatment of the GL2 component, which requires itself some expertise to be handled. Nevertheless, several tricks and improvements can be used to make the OEP calculations simpler and more reliable,^103^ thus various upgrades can be easily seen from the practical point of view for the SCF–ACM method. Anyway, these are left for future works, as in this paper we wanted to focus only on the core of problem without adding too many technical details.

Having been able to perform SCF ACM calculations on various systems, we could perform a thorough assessment of the functionals, finding important results. For strongly correlated systems, such as the harmonium atom and the hydrogen molecule at the dissociation limit, the ACM SCF calculations yield very accurate results taking advantage of the incorporated strong-correlation limit and also thanks to the novel hPC functional for *W*_∞_ and *W*_∞_^′^ that proved to be very accurate for these cases. For molecular systems, we found that the overall accuracy using SCF orbitals depends on the quality of the underlying ACM, in line with the refs (24) and (25). In any case, the ISI-hPC yields already quite correct SCF potentials and total energies: nevertheless, its accuracy needs to be further verified for reactions and atomization energies.

Thus, we can finally conclude that, despite some limitations, the overall accuracy of the ISI functional (and partially also of the SPL one), when the full SCF solution is taken into account, is overall satisfactory, especially considering the following: (i) it does not employ any parameter obtained from molecular systems, and (ii) the approach is within a pure KS formalism with a local potential. These results and the availability of a working SCF procedure for general ACM formulas now open to the application and testing on other systems beyond the simple ones considered in this work. Moreover, it paves the path toward the development of more accurate ACM functional forms (see e.g. ref (46)) as well as to further development of *W*_∞_ and *W*_∞_^′^ approximations, with improved accuracy for molecular systems.

## Interpolation Formulas

In the following, we report the ISI and SPL interpolation formulas.

ISI formula^38^
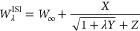
15with
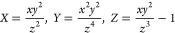
16
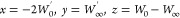
17which yields for the XC energy
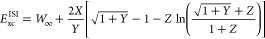
18

SPL formula^40^
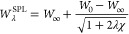
19with
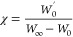
20

The SPL XC functional reads
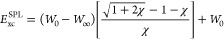
21

Note that this functional does not make use of the information from *W*_∞_^′^.

## Supporting Information Available

Details on the basis set, dissociation curve of *H*_2_ with the SPL functional, and further results for Hooke’s atom (PDF)
