# Supplementary material for: Self-Consistent Implementation of Kohn–Sham Adiabatic Connection Models with Improved Treatment of the Strong-Interaction Limit
Source: J Chem Theory Comput. 2022 Sep 12;18(10):5936–47. doi: 10.1021/acs.jctc.2c00352 (PMC9558377; doi:10.1021/acs.jctc.2c00352)
Supplement: Supplementary file 1 — ct2c00352_si_001.pdf [file ct2c00352_si_001.pdf]

Supporting information to "Self-Consistent Implementation of Kohn-Sham Adiabatic Connection Models with Improved Treatment of the Strong-Interaction Limit"

Szymon Śmiga[a], Fabio Della Sala[b,c], Paola Gori-Giorgi[d], Eduardo Fabiano[b,c]

[a] *Institute of Physics, Faculty of Physics, Astronomy and Informatics, Nicolaus Copernicus University, Grudziadzka 5, 87-100 Torun, Poland*

[b]*Institute for Microelectronics and Microsystems (CNR-IMM), Via Monteroni, Campus Unisalento, 73100 Lecce, Italy*

[c]*Center for Biomolecular Nanotechnologies@UNILE, Istituto Italiano di Tecnologia (IIT), Via Barsanti, 73010 Arnesano (LE), Italy*

[d]*Department of Chemistry & Pharmaceutical Sciences and Amsterdam Institute of Molecular and Life Sciences (AIMMS), Faculty of Science, Vrije Universiteit, De Boelelaan 1083, 1081HV Amsterdam, The Netherlands,*

E-mail: szsmiga@fizyka.umk.pl

August 19, 2022

Contents

1 Summary of the basis sets 2

2 Dissociation of H_2 with SPL functional 2

3 Hookes atom results 3

1 Summary of the basis sets

- **He, He$_2$**: even tempered $20s10p2d$ basis.
- **Be, Ne, Ne$_2$**: uncontracted ROOS-ATZP basis[1].
- **Mg**: uncontracted aug-cc-pVTZ basis set[2].
- **Ar**: s and p basis functions from the uncontracted ROOS-ATZP[1] basis set and d and f functions from the uncontracted aug-cc-pwCVQZ basis set[3].
- **HF, CO, H$_2$O, H$_2$, Cl$_2$, N$_2$, HCl, NH$_3$, C$_2$H$_6$**: uncontracted cc-pVTZ basis set of Dunning[4].

2 Dissociation of H$_2$ with SPL functional

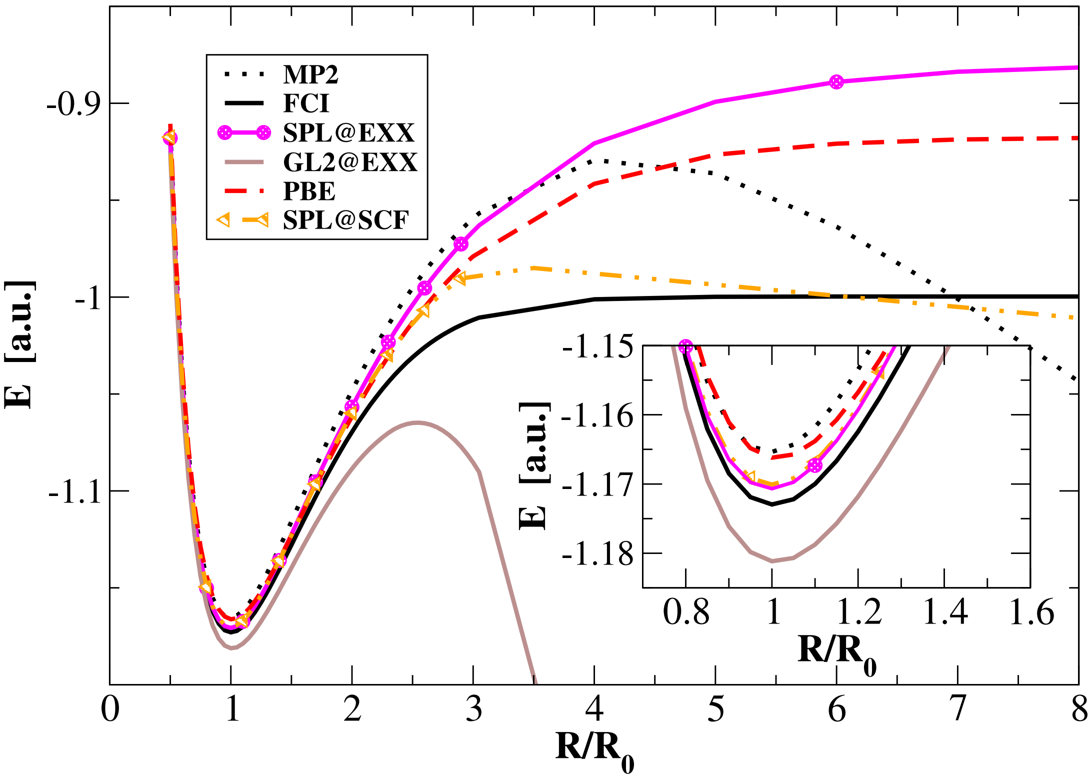

Figure S1: The total energy of the H$_2$ molecule as it is stretched calculated with the various methods. The inset presents the same data around the equilibrium distance.

3 Hookes atom results

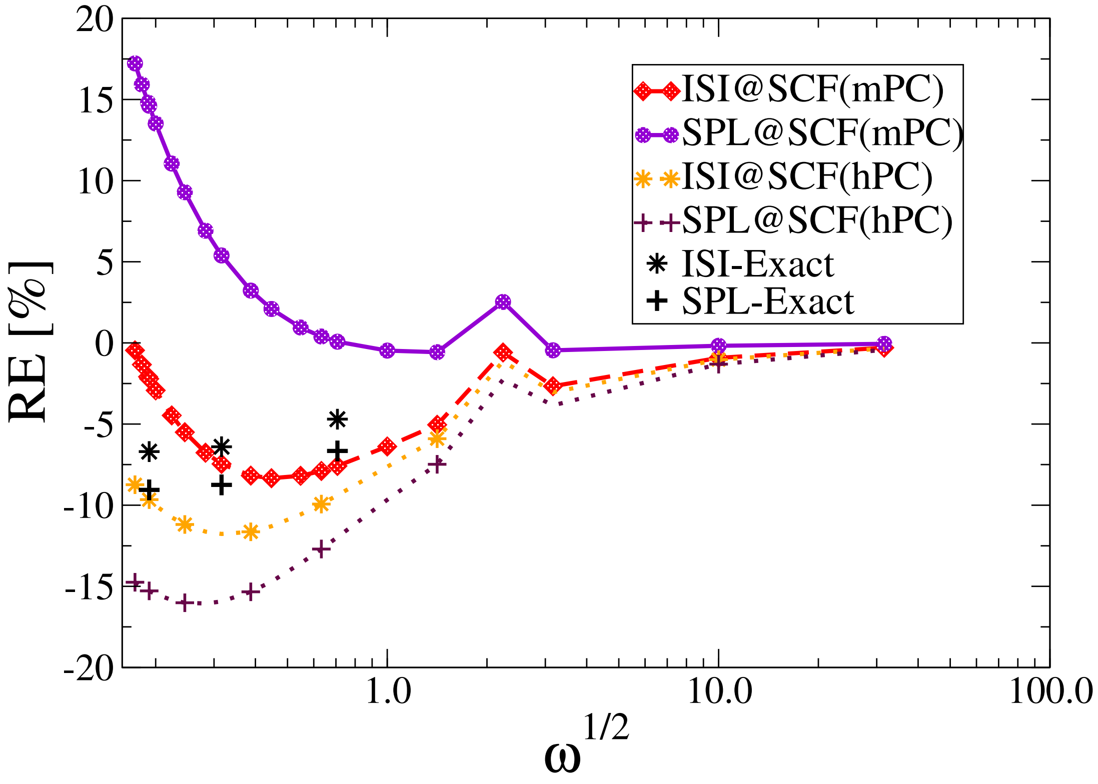

Figure S2: Relative error on correlation energies of harmonium atoms for various values of ω computed at @SCF orbitals for ISI and SPL functionals using the hPC and mPC models for the strong-interaction functionals. The errors have been computed with respect FCI data obtained in the same basis set[5]. The exact ISI and SPL values are taken from Ref.[6], and are obtained by inserting exact densities into the ISI and SPL functionals, including the exact treatment (SCE) of the strong-interaction limit.

References

[1] Per-Olof Widmark, Per-Åke Malmqvist, and Björn O Roos, "Density matrix averaged atomic natural orbital (ano) basis sets for correlated molecular wave functions", *Theor. Chim. Acta* **77**(5), pp. 291–306 (1990).

[2] D. E. Woon and T. H. Dunning, Jr., "Gaussian basis sets for use in correlated molecular calculations. III. The atoms aluminium through argon", *J. Chem. Phys.* **98**, pp. 1358–1371 (1993).

[3] K. A. Peterson and T. H. Dunning, Jr, *J. Chem. Phys.* **117**, pp. 10548 (2002).

[4] Thom H. Dunning, "Gaussian basis sets for use in correlated molecular calculations. i. the atoms boron through neon and hydrogen", *J. Chem. Phys.* **90**(2), pp. 1007–1023 (1989).

[5] Eduard Matito, Jerzy Cioslowski, and Sergei F. Vyboishchikov, "Properties of harmonium atoms from fci calculations: Calibration and benchmarks for the ground state of the two-electron species", *Phys. Chem. Chem. Phys.* **12**, pp. 6712–6716 (2010).

[6] Derk P Kooi and Paola Gori-Giorgi, "Local and global interpolations along the adiabatic connection of dft: a study at different correlation regimes", *Theor. Chem. Acc.* **137**(12), pp. 166 (2018).
